# Supplementary material for: Telomere analysis using 3D fluorescence microscopy suggests mammalian telomere clustering in hTERT-immortalized Hs68 fibroblasts
Source: Commun Biol. 2019 Dec 4;2:451. doi: 10.1038/s42003-019-0692-z (PMC6893014; doi:10.1038/s42003-019-0692-z)
Supplement: Supplementary file 1 — Supplementary Information [file 42003_2019_692_MOESM1_ESM.pdf]

I. Fluorescence *in situ* hybridization (FISH) staining

- Perform metaphase spread derived from cells of interest
  - Optimize PNA concentration and staining conditions
  - Confirm telomere count
- Seed cells of interest on coverslips
  - Stain interphase cells with PNA probe (TelC-647) and DAPI

II. Image Acquisition

- Capture raw image
  - Z-stack of sample using full FOV of camera
  - Multiple wavelengths (DAPI, Telomere)
- Capture dark frame (9-16 frames)
  - Using same settings as raw image
  - Shutter closed
- Capture flatfield image (9-16 frames)
  - Using same settings as raw image
  - Plastic slide or uniform fluorescent sample

III a. Darknoise and flatfield correction

- Import acquired images into ImageJ
  - Split channels and convert to TIFF
  - Use 'Process -> Image Calculator' to carry out the following calculations
  - Apply to entire Z-stack
- Dark noise correction
  - Subtract both flatfield image and raw image by darkframe
- Flatfield correction
  - Divide darknoise-corrected raw image by darknoise-corrected flatfield image
  - Apply 32-bit floating
- Scale intensities to a 12-bit range
  - Multiply the corrected image by a constant value
  - Save as TIFF

Supplementary Figure 1. Detailed description and stepwise protocol of the methods described in this paper (continued on the next page).

III b. Huygens Deconvolution

- Import corrected images into Huygens Essential
- Set general, optical and channel parameters
  - Fill in imaging and sample parameters
  - Set all parameters to verified
- Start deconvolution wizard
  - Background value was automatically calculated
  - Use Classic Maximum Likelihood Estimation (CMLE) for deconvolution algorithm
  - Correct original for bleaching and unstable illumination
  - Save as Delta Vision r3d format

IV a. ImageJ 2D

- Import deconvolved images (TelC-647)
- Create a maximum intensity projection
  - Image -> Stacks -> Z-project
- Count telomeres of individual cells
  - Create Region of Interest for individual cells
  - Process -> Find Maxima
  - Use different noise tolerances to determine expected telomere count
  - Confirm expected telomere count with metaphase spread

IV b. Imaris 3D

- Import deconvolved image (TelC-647)
- Start ‘Spots’ wizard (see video)
  - Choose ‘Different spot sizes (region growing)’
  - Fill in estimated XY and Z diameter (Model PSF-elongation along Z-axis) based on manual measurements (see video)
  - Enable ‘Background subtraction’
  - Choose ‘Quality’ for Filter Type
  - Spots regions from Absolute Intensity
  - Choose ‘Intensity Threshold’ based on accurate representation of telomeric signal
  - Diameter from Region Border
- Color-code spots based on any feature
- Export all statistics to Excel-file

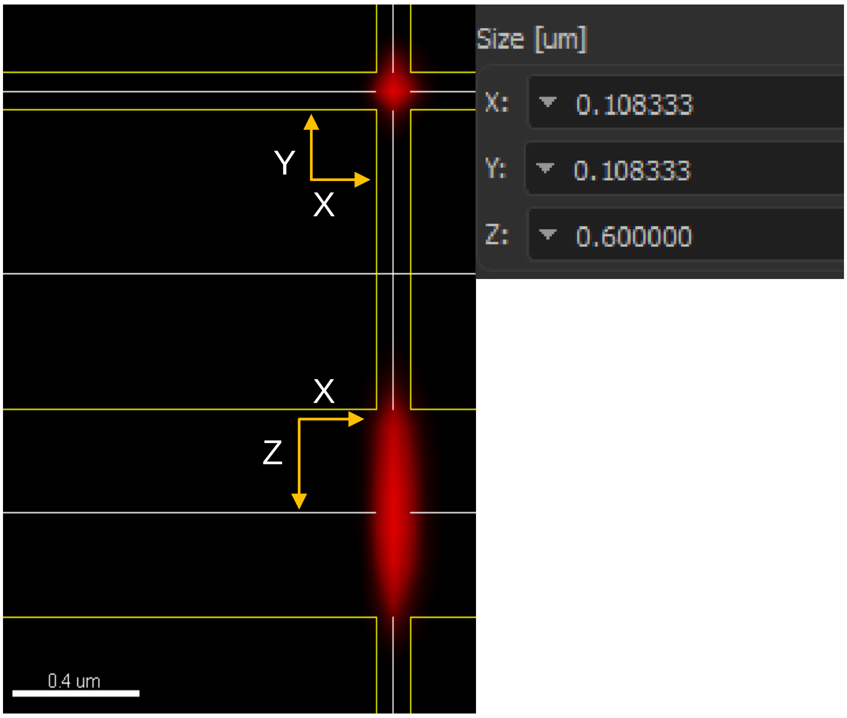

Supplementary Figure 2. Visualization of the point spread function of a deconvolved, wide-field image of one 100 nm bead (red) in Imaris. See Supplementary Table 3 for the summary of the calculations based on 100 nm beads.

A

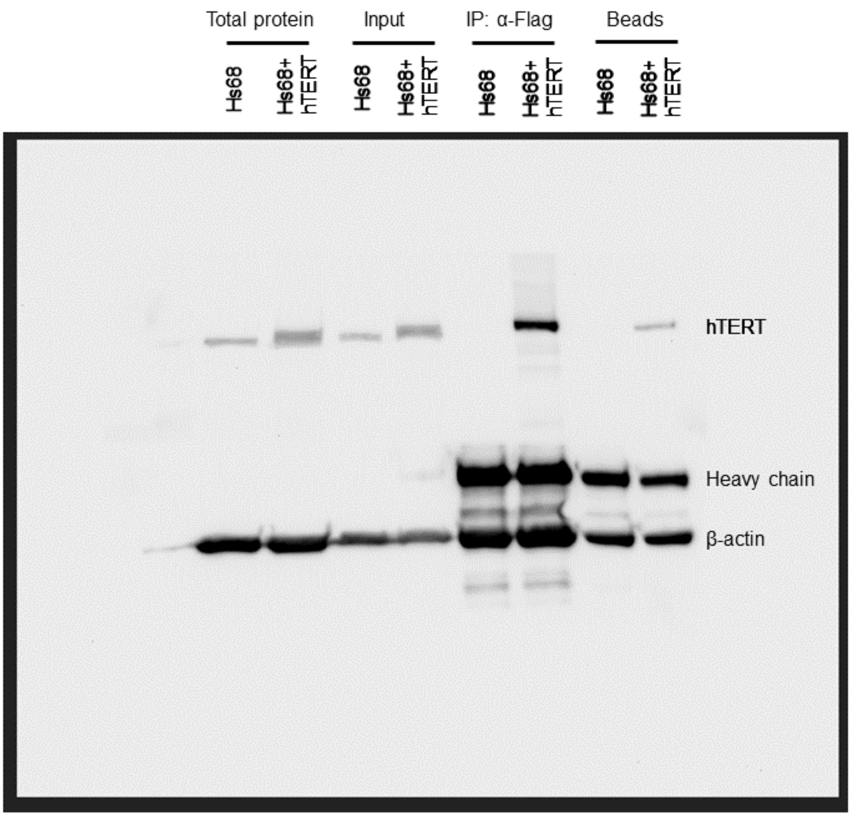

B

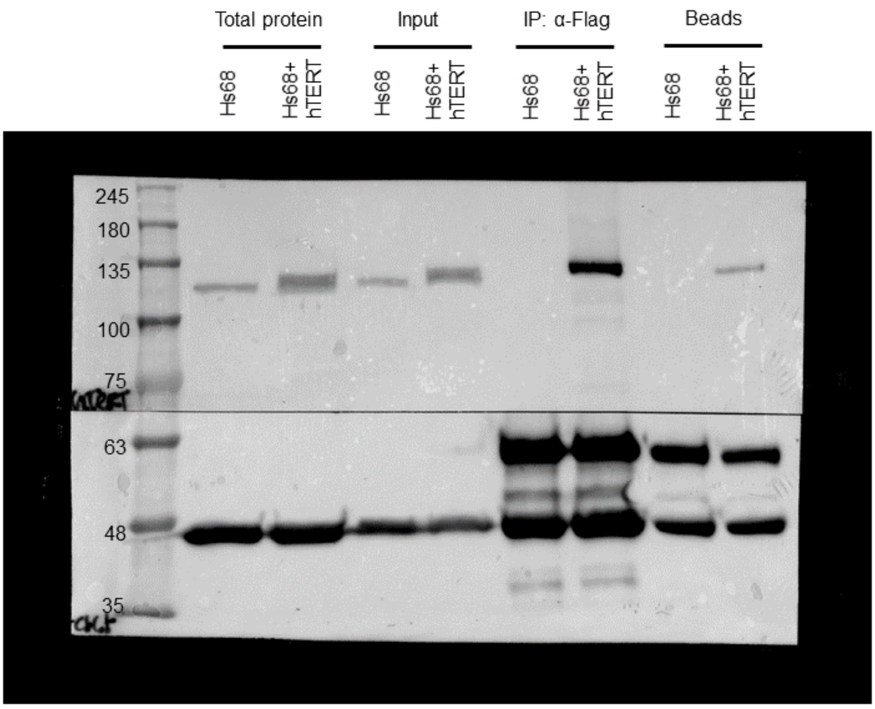

Supplementary Figure 3. Full uncropped blot images of hTERT expression in Hs68 and Hs68+hTERT (Figure 6A). A) Chemiluminescence image of hTERT and β-actin protein levels. B) Overlay of colorimetric image with the chemiluminescence image.

A

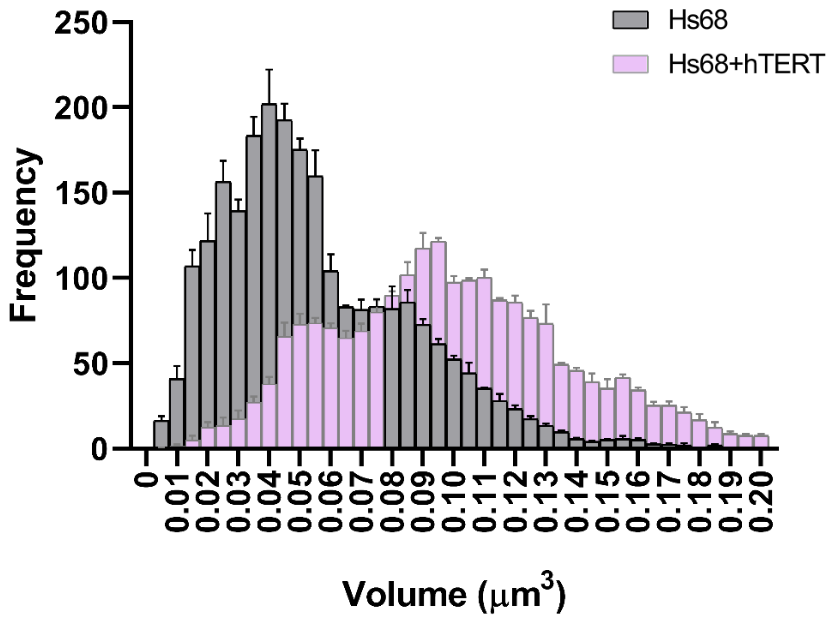

B

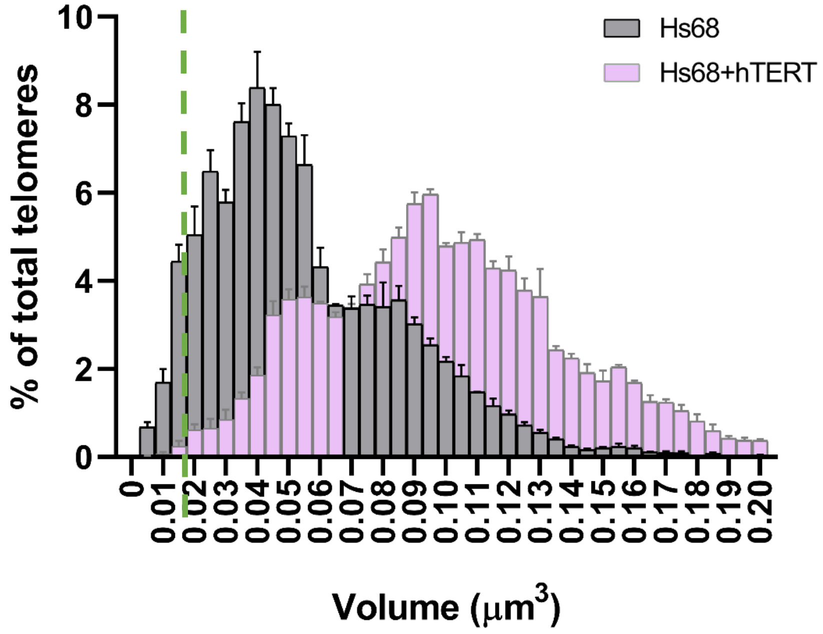

Supplementary Figure 4. Telomere volumes measured in Hs68 and Hs68+hTERT. A) Frequency distribution of the volume of the individual telomeres in Hs68 and Hs68+hTERT cells. B) Histogram representing the percentage of total telomeres within each volume bin. Green dotted line separates spots smaller than the measured diffraction-limited volume (≤ 0.015 μm^3) from the rest. See Supplementary Table 3 for calibration of measured diffraction-limited volume. Error bars = SD, n=3 biologically independent experiments, >30 cells per n. See Supplementary Data 4 for source data.

Spots	Hs68	Hs68+hTERT	Ratio (Hs68+hTERT ÷ Hs68)
**INTENSITY (average)**			
Intensity Center	29391 ± 6535	87085 ± 18935	2.99 ± 0.46
Intensity Sum	593587 ± 114011	2342980 ± 403581	3.95 ± 0.54
Intensity Mean	10712 ± 1684	24207 ± 3999	2.27 ± 0.26
**SIZE (average)**			
Diameter X,Y (µm)	0.311 ± 0.004	0.392 ± 0.002	1.26 ± 0.01
Area (µm^2)	0.778 ± 0.018	1.218 ± 0.008	1.57 ± 0.03
Volume (µm^3)	0.053 ± 0.002	0.102 ± 0.001	1.92 ± 0.05

Supplementary Table 1. Average of individual telomere measurements in Imaris. Numbers reflect the average per biological repeat ± the standard deviation (SD). n=3, >30 cells per n. See Supplementary Data 4 for source data.

Average CV in %	Hs68	Hs68+hTERT
**INTENSITY**		
Intensity Center	35.1	42.7
Intensity Sum	39.1	48.1
Intensity Mean	24.5	33.5
**SIZE**		
Diameter X,Y (µm)	5.7	6.0
Area (µm^2)	11.4	11.8
Volume (µm^3)	17.0	17.4

Supplementary Table 2. Average coefficient of variation (CV) is calculated by taking the mean telomere values per cell per biological repeat and dividing that by their calculated SD. The ratio is multiplied by 100% to show the variation as a percentage. See Supplementary Data 4 for source data.

	Beads
Diameter (nm)	100
Volume sphere (x 10^6 nm^3)	4.19
Volume sphere (μm^3)	0.00419
Pixel size (nm)<br>**no diffraction considered*	108
Step size (nm)	100
Theoretical voxel size (μm^3)<br>**no diffraction considered*	0.001166
Diffraction limited diameter X,Y (nm)	~ 200
Diffraction limited diameter Z (nm)	~ 600
Diffraction limited volume (μm^3)<br>**Theoretical smallest volume*<br>*$V_{ellipsoid} = \frac{4}{3}\pi\left(\frac{x}{2}\cdot\frac{y}{2}\cdot\frac{z}{2}\right)$	0.0126
Spot Volume (intensity = 800)<br>**calculated by Imaris (μm^3)*<br>**n=300 beads, ± SD*	0.01586 ± 0.001

Supplementary Table 3. Imaris calibration and 3D measurements on deconvolved wide-field images of 100 nm beads.

	Hs68	Hs68+hTERT
Average telomere count/cell<br>**Imaris 3D spots*	73	64
Predicted # of spots containing >1 telomere<br>**Assuming 92 telomeres/cell*	19	28
Predicted # of singular telomeres/cell	54	36
% of spots that represents singular telomeres	$\frac{54}{73} * 100 = 74\%$	$\frac{36}{64} * 100 = 56\%$
Volume representing singular telomeres<br>**See Supplementary Figure 4*	≤0.07 μm^3	≤0.10 μm^3

Supplementary Table 4. Summary of the count and size of the spots analyzed in Imaris and the predicted amount of spots that represents singular telomeres.

	Hs68	Hs68+hTERT
Nuclear volume (µm^3)	641.1	609.4
Sum telomere volume per cell (µm^3)	4.0	6.8
% nucleus occupied by telomeres	0.64	1.16

Supplementary Table 5. Average measured telomere and nuclear volume in Hs68 and Hs68+hTERT. n=1 biologically independent experiment, >30 cells. See Supplementary Data 4 for source data.
