# Supplementary material for: Telomere analysis using 3D fluorescence microscopy suggests mammalian telomere clustering in hTERT-immortalized Hs68 fibroblasts
Source: Commun Biol. 2019 Dec 4;2:451. doi: 10.1038/s42003-019-0692-z (PMC6893014; doi:10.1038/s42003-019-0692-z)
Supplement: Supplementary file 2 — Description of additional supplementary files [file 42003_2019_692_MOESM2_ESM.pdf]

Description of additional supplementary files

Supplementary Data 1. Source data used for graphs shown in Figure 4.

Supplementary Data 2. Source data used for graphs shown in Figure 6.

Supplementary Data 3. Source data used for graphs shown in Figure 7.

Supplementary Data 4. Source data used for graphs shown in Supplementary Figures and Tables.
